# Cross-Domain TransNet for sparse-view CT reconstruction

**DOI:** 10.3389/fnume.2026.1711533

**Published:** 2026-07-06

**Authors:** Junling Wang, Chunhua Zou, Hongjie Yang, Peixi Liao, Xiaojun Mo, Yingcui Gao, Yanan Ning, Jiahao Lai

**Affiliations:** 1 Department of Radiology, Chengdu Sixth People's Hospital, Chengdu, China; 2 Department of Radiology, The First People’s Hospital of Chengdu, Chengdu, China; 3 School of Information and Software Engineering, University of Electronic Science and Technology of China, Chengdu, China; 4 Department of Stomatology, Chengdu Sixth People's Hospital, Chengdu, China; 5 Department of Radiology, General Hospital of the Western Theater Command of the Chinese People's Liberation Army, Chengdu, China; 6 Department of Interventional Radiology, Sun Yat-Sen Memorial Hospital, Sun Yat-Sen University, Guangzhou, China

**Keywords:** CT reconstruction, dual-domain, self-attention, sparse-view CT, transformer

## Abstract

**Introduction:**

Sparse-view computed tomography (CT) reconstruction is crucial for clinical diagnostics, as reducing radiation exposure is essential to minimize risks to patients. Existing dual-domain reconstruction methods leverage both image and projection domains but often process them sequentially, overlooking their implicit correlations.

**Methods:**

To address this limitation, we propose Cross-Domain TransNet, a Transformer-based dual-domain framework for sparse-view CT reconstruction. The proposed model captures long-range dependencies within each domain and integrates image and sinogram representations through a hybrid self-attention mechanism. In addition, a Convolution Fusion Layer (CFL) is introduced to enhance feature interactions and facilitate more effective utilization of dual-domain information.

**Results:**

Extensive experiments on the NIH-AAPM dataset demonstrate the superior performance and generalization capability of the proposed method under various sparse-view settings. The results show that Cross-Domain TransNet consistently improves reconstruction quality, effectively suppresses noise, and reduces artifacts, outperforming both conventional reconstruction algorithms and state-of-the-art deep learning approaches.

**Conclusion:**

Cross-Domain TransNet provides an effective and robust solution for sparse-view CT reconstruction. By fully exploiting complementary information from both image and projection domains, the proposed framework enhances diagnostic image quality while supporting radiation dose reduction.

## 1 Introduction

Computed Tomography (CT) has become an indispensable tool in clinical diagnostics, playing a critical role during the COVID-19 pandemic by enabling rapid screening and assessment of disease severity (1, 2). However, increasing reliance on CT has raised concerns regarding radiation-induced health risks. Numerous studies have demonstrated that ionizing radiation from CT scans is associated with elevated cancer risk in both adults and children (3–5). For instance, exposure has been linked to higher incidences of leukemia and brain tumors, correlating with radiation doses to bone marrow and brain tissue (6). A long-term study of 31,462 patients reported that CT-related exposure increased lifetime cancer risk by 0.7% across the population and overall cancer mortality by 1%, with risks rising to 2.7%–12% in patients undergoing multiple scans (7). Moreover, children are particularly vulnerable, being three to four times more sensitive to radiation than adults, which may lead to more severe long term health effects (8).

In recent years, Sparse-View CT (SVCT) has gained wide-spread attention in clinical application, as its potential to reduce patient radiation exposure by lowering x-ray doses. However, lowering x-ray doses often leads to increased noise and artifacts in the reconstructed CT images, severely compromising image quality and affecting clinical diagnosis. Mitigating the adverse effects of reduced radiation doses in CT image reconstruction has therefore become a key focus of current research. Deep learning (DL), as an emerging artificial intelligence technology, has been demonstrated remarkable potential in the field of image processing and analysis (9). Numerous studies have confirmed the feasibility and effectiveness of using DL methods for sparse-view CT image reconstruction, yielding significant improvements in image quality over traditional approaches. As a result, employing DL methods for sparse-view CT image reconstruction is of significant research importance and offers promising practical applications. By reducing patient radiation exposure, enhancing the accuracy of medical image diagnostics, and advancing medical imaging technology, DL methods can provide safer, more precise, and higher-quality reconstructed medical images for clinical practice. This advancement ultimately contributes to reducing radiation exposure and enhancing the healthcare quality of patients.

By now, the advanced methods for the reconstruction of sparse-view CT images can be summarized into two main categories. Traditional methods typically involve minimizing a unified objective function iteratively to reconstruct images from noisy sinogram data. This objective function integrates prior information of the image domain with the statistical properties of the sinogram data. Total variation (TV) and its variants (10), non-local means (11), and dictionary learning (12) are image priors commonly used in recent researches. While these methods have significantly improved reconstruction quality, they depend on iterative forward and backward projection operations, requiring detailed knowledge of the specific scanner geometry. This results in high computational complexity and longer reconstruction times. In recent years, DL methods have achieved significant breakthroughs in sparse-view CT reconstruction tasks. These researchers utilize DL network structures such as Convolutional Neural Networks (CNNs) to acquire the mapping relationship between sparse-view projection data and high-quality CT images. DL techniques can effectively reduce noise, recover details during the reconstruction process and lead to substantial improvements in image quality through automatically learning complex image features from large data.

Current deep learning (DL) methods for sparse-view CT reconstruction can be broadly categorized into three groups, with CNN and U-Net based architectures being the most dominant. Representative works include RED-CNN (13), which combines deconvolution, autoencoders, and residual connections; DenseNet with deconvolution (14) to enhance image details; FCPRN (15), which integrates FCN with pyramidal residual blocks for multi-scale feature extraction; SR-CNN (16), designed to preserve edges and fine structures; and RNN-based models (17) for stripe artifact removal. Yan et al. further proposed TLD-CDL (18), combining convolutional dictionary learning, transfer learning, and dense connections for robust feature representation. Most of these DL methods operate in the image domain, as direct access to projection data is often impractical. Typically, sparse-view data are first reconstructed using filtered back projection (FBP), followed by artifact reduction via neural networks. However, this sequential strategy cannot recover information lost in projection data, limiting the potential of image-only approaches (19, 20).

It often results in severe streak artifacts and diminished image quality by applying traditional image reconstruction algorithms like FBP directly to sparse-view projection data. To address this issue, neural networks are employed to fill in the missing data in the projection domain, after which the completed projection data can be used in the next image reconstruction process. Liu et al. proposed a generative adversarial network (GAN) known as Pix2Pix to perform pixel-level reconstruction for sinogram data (21). Compared to traditional linear interpolation methods, Pix2Pix performed better while reconstructing high quality images. Kim et al. introduced an unsupervised training method based on deep neural networks in the projection domain (22). In which, it recovers sparse-view projection data and achieves performance comparable to supervised methods without full dose CT images. Besides, to address metal artifacts in CT, Ghani et al. proposed a framework named Deep-MAR (23). This method is based on GANs and treats projection data corresponding to dense metal regions as missing data.

Repairing data in the projection domain can mitigate signal loss due to sparser radiation doses, which enables the reconstruction process to commence under less noise conditions. Consequently, projection domain processing methods are more robust in handling noise. However, these methods require detailed knowledge of the CT device's hardware parameters typically. The wide variety of equipment manufacturers and models makes it hard to obtain and match these hardware parameters accurately, thereby limiting the practical applicability of these approaches. Moreover, in the three projection domain processing methods mentioned earlier, FBP is still used for direct reconstruction after repairing the completed projection data. To some extent, when DL methods are only employed for projection domain data repair, the limitations of traditional methods may still affect the reconstruction outcomes during image post-processing. Compared with single-domain deep learning methods, most current sparse-view CT reconstruction studies focus on dual domain processing, which simultaneously recovers projection data and reconstructs image data. Representative works include DuDoNet (24), which employs a mask pyramid U-Net for projection preprocessing and image post-processing; DuDoTrans (25), which integrates Swin-Transformer and CNN to repair sinograms; CAGAN (26), which combines adversarial autoencoders with coordinate attention; DuDoUFNet (27), which suppresses metal artifacts in the projection domain and reduces noise in the image domain; and DRONE (28), which embeds, refines, and enhances features to improve image quality. These approaches typically adopt a sequential workflow: first repairing projection data, then reconstructing CT images using algorithms such as FBP, followed by image domain refinement. While this improves quality over single domain methods, it essentially couples two independent processes without fully exploiting correlations between domains. Moreover, repeated use of FBP may introduce additional noise and artifacts, limiting reconstruction performance.

Recent progress in implicit neural representations has provided new solutions for sparse-view CT reconstruction by representing images or volumes as continuous functions optimized via coordinate-based multilayer perceptrons. For instance, NeRP (29) integrates patient-specific prior images into the network and optimizes this implicit representation under projection constraints, enabling robust reconstruction in highly sparse scenarios when longitudinal priors are accessible. TomoGRAF (30) extends NeRF-style volumetric rendering by embedding x-ray imaging physics into a generative radiance field. It achieves better geometric consistency in very sparse settings, including cases with only one or two views, through differentiable volume rendering that combines both projection and volumetric supervision. These methods based on implicit neural representations offer notable data efficiency, seamless integration of imaging physics, and the ability to produce structurally coherent reconstructions under severely limited data conditions. However, they also come with certain limitations, such as reliance on patient-specific priors in approaches like NeRP, high computational costs due to test-time optimization or volumetric rendering, and reported challenges in accurately recovering CT numbers and fine anatomical details under extreme sparsity.

In contrast, a transformer-based formulation adopts a different modeling strategy compared to the above methods. It learns global dependencies across projection views rather than explicitly parameterizing volumes or depending on patient-specific priors. Once trained, the model enables fast feed-forward reconstruction without requiring test-time optimization. Its global attention mechanism facilitates effective integration of information from sparse views and supports robust generalization. As a result, the transformer-based approach serves as a complementary alternative to the above techniques, which continue to excel in leveraging geometric priors and patient-specific information, especially under extremely sparse acquisition conditions.

## 2 Materials and methods

### 2.1 Datasets

The training and testing of the method used publicly available data from the 2016 NIH-AAPM-Mayo Low Dose CT Grand Challenge dataset (31), denoted as Mayo dataset. Besides, a scanning geometry with a fan-beam x-ray source comprising 800 detector elements was simulated following the settings described in references (32–34). Specifically, for this study, a total of 2,378 3 mm slices reconstructed using the B30 reconstruction kernel were chosen from 10 patients, with a resolution of 256 × 256. Out of these, 237 slices were randomly selected for testing, while the remaining 2,141 slices were used for training and validation for the models. The details were introduced in Supplementary Materials S1.

### 2.2 Cross-Domain TransNet architecture

**Figure 1:**
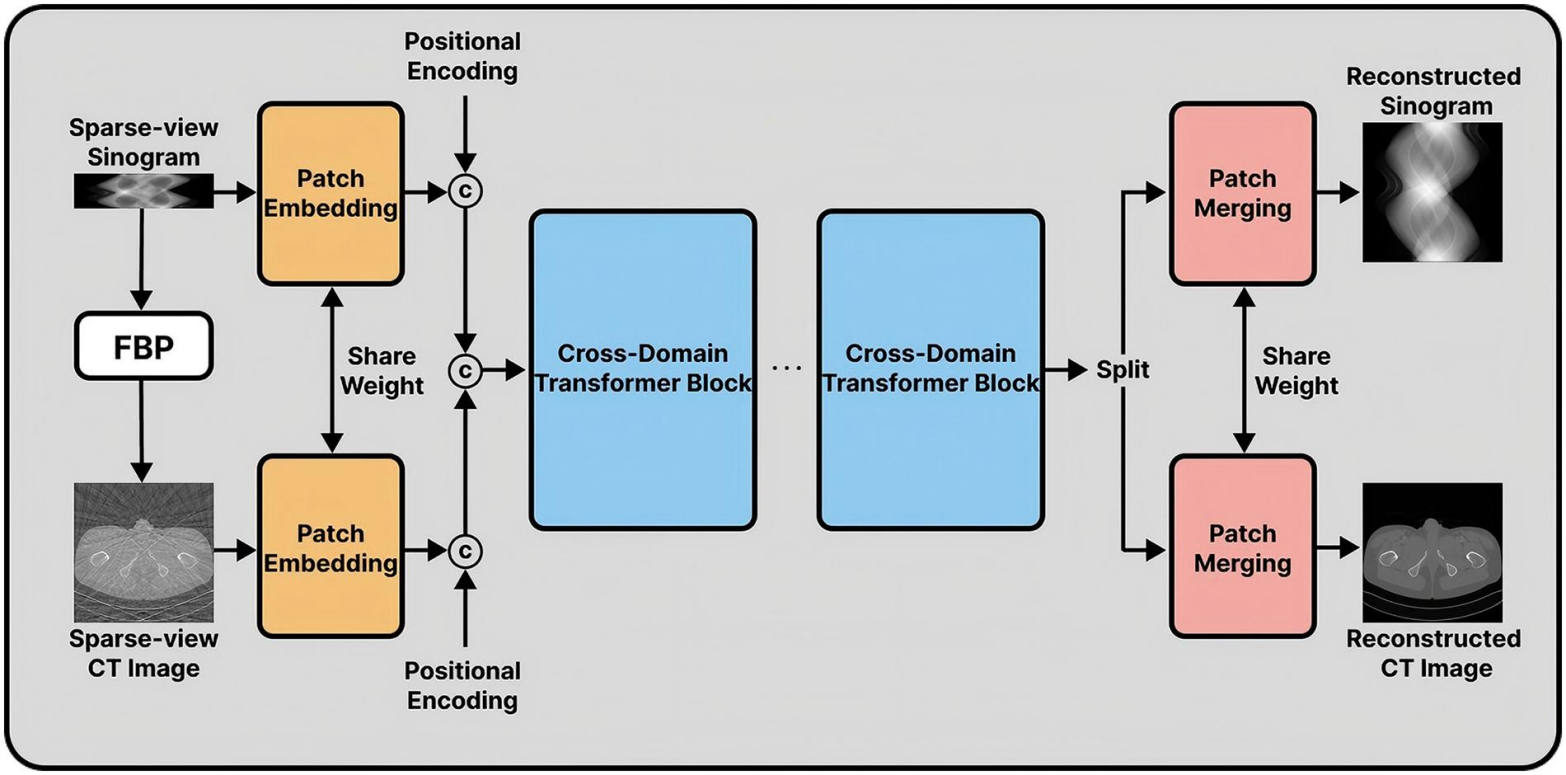
Framework of CDTN. The overall framework of the method proposed is primarily composed of three parts: initial Patch Embedding operation, stacked Cross-Domain Transformer modules, and Patch Merging operation.

**Figure 2:**
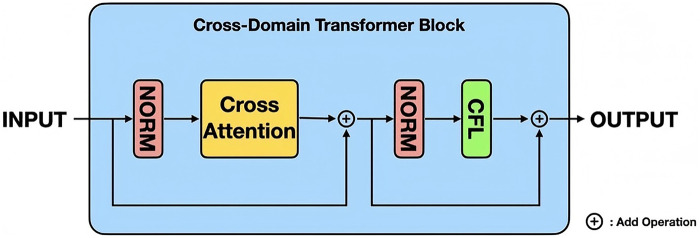
Architecture of cross-domain transformer block. The Cross-Domain Transformer Block takes concatenated dual-domain tokens as input, first applying layer normalization to standardize the tokens and prevent data offset during training.

**Figure 3:**
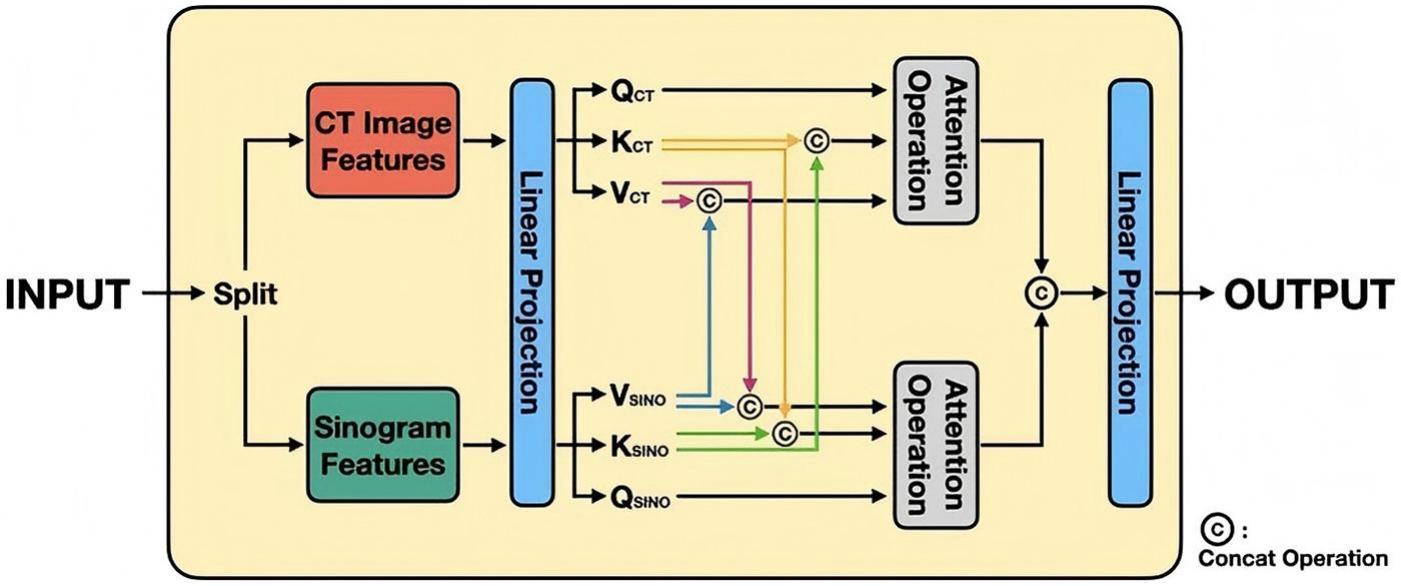
Architecture of cross attention operation. The cross-attention module then splits the dual-domain tokens into single-domain tokens, where self-attention within each domain is synchronized with cross-attention between the domains.

**Figure 4:**
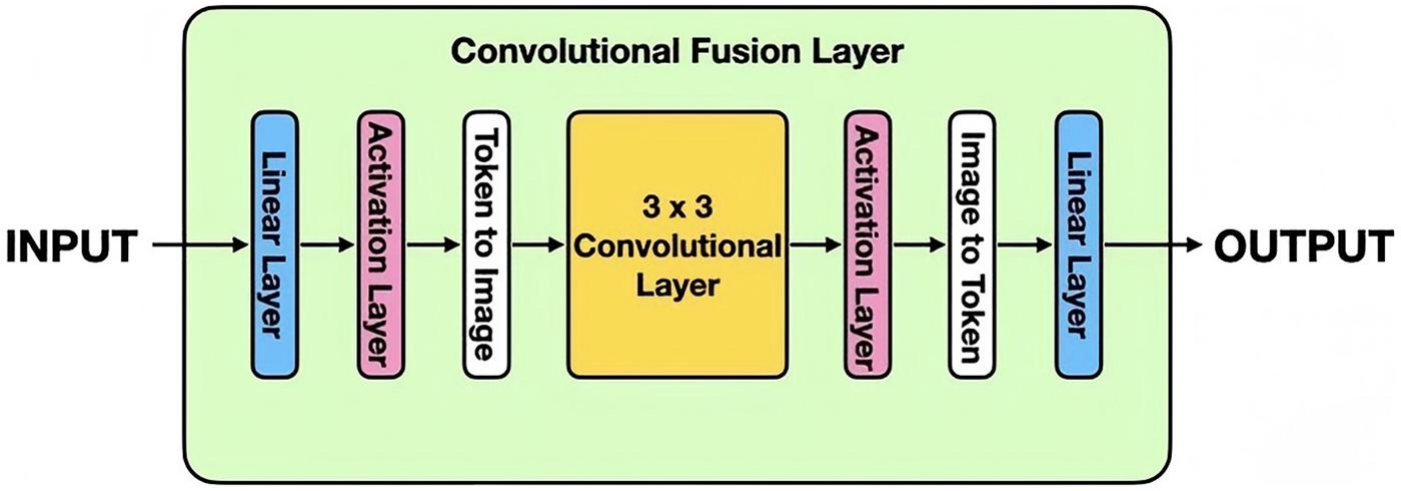
Architecture of convolutional fusion layer (CFL). The CFL first maps input tokens into a spatial feature map through a linear projection and activation. A convolution layer is then applied to perform local feature fusion. The fused features are subsequently mapped back to the token representation via another activation and linear layer. This module enables seamless interaction between the token space and image space, allowing the model to exploit both global transformer representations and local convolutional priors.

We proposed a novel sparse-view CT reconstruction method based on the Vision Transformer architecture that incorporates a dual-domain cross-attention mechanism (Figure 1). The main idea is to progressively extract potential relationships between image domain and projection domain data, thereby complementing and enhancing the recovery of both domains. The method is primarily composed of three parts: initial Patch Embedding operation, stacked Cross-Domain Transformer modules (Figures 2, 3), and Patch Merging operation (Figure 4). Our model builds upon the previous models and incorporates new fusion ideas, enabling cross-domain information to be deeply integrated, thereby achieving better reconstruction results. The framework was introduced in Supplementary Materials S2 in details.

### 2.3 Dual-domain loss function

This work applied a dual-domain loss function to achieve better reconstruction results. The dual-domain loss function comprises three components: image reconstruction loss, image perceptual loss, and reconstruction projection consistency loss. The details were shown in Supplementary Materials S2.

### 2.4 Experimental setup

In this section, we implemented the proposed method in Python using the PyTorch library and employed the Adam (35). The details of our experimental setup were shown in Supplementary Materials S3.

### 2.5 Evaluation

For metrics, we adopted PSNR, SSIM, and NRMSE as basic evaluation metrics. Besides, we further conducted histogram-based quantitative analyses to evaluate the intensity consistency and residual error distribution of different reconstruction methods. For each test case, the full-view reconstruction was used as the reference image, and the reconstructed image obtained by each sparse-view method was compared with it on a pixel-wise basis.

## 3 Results

**Figure 5:**
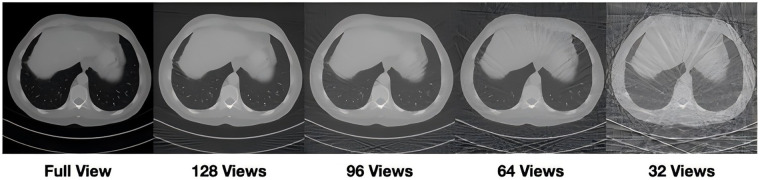
A set of 5 simulated images from different views. It displays a set of image data from the 3mmB30 dataset. It is evident from the images that as the radiation dose in the projection data decreases, the noise and streak artifacts in the reconstructed images produced by the FBP method gradually increase.

A set of image data from the 3mmB30 dataset was displayed in Figure 5, including regular dose data and simulated noise of varying intensities. These data were reconstructed using the FBP algorithm to generate CT images. It is evident from the images that as the radiation dose in the projection data decreases, the noise and streak artifacts in the reconstructed images produced by the FBP method gradually increase.

### 3.1 Comparison

We compared the proposed method with three recent DL-based approaches: RED-CNN (13) and CNCL (36) in the image domain, and DuDoTrans (25) in the dual-domain. Specifically, RED-CNN is the first to introduce convolutional neural networks into low-dose CT image post-processing, making significant progress in denoising compared to traditional methods. CNCL is a method based on generative adversarial networks. DuDoTrans is a dual-domain processing method based on an improved Transformer framework. FBP is widely used in routine-dose CT reconstruction in clinical practice due to its low computational resource requirements and fast speed. FBP is widely used in routine-dose CT reconstruction in clinical practice due to its low computational resource requirements and fast speed. Besides, we used official or widely adopted open-source implementations and followed the original parameter settings, using the same dataset for training, validation, and testing.

### 3.2 Visualization results

**Figure 6:**
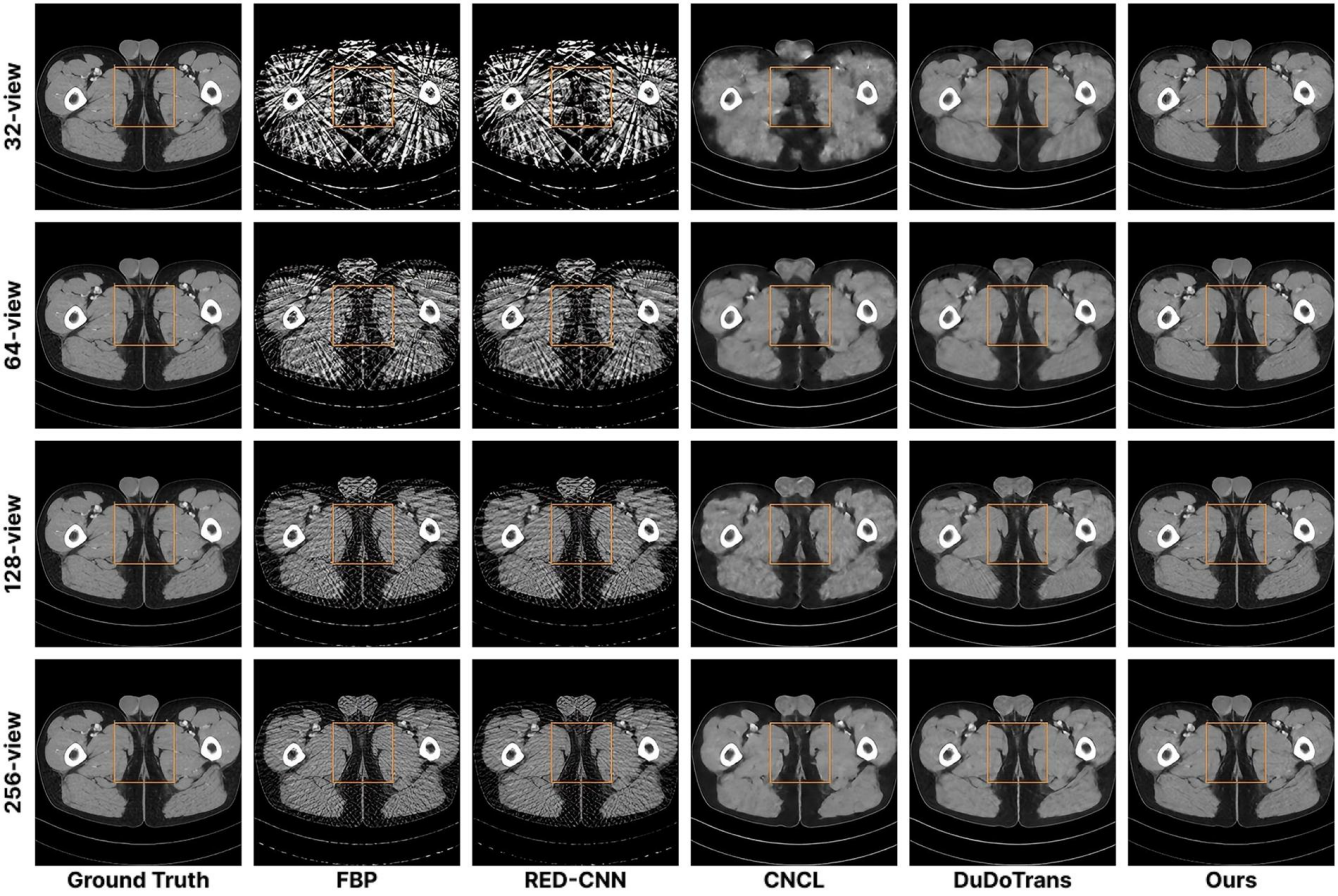
Qualitative comparison on mayo dataset, each row from top to bottom corresponds to 32, 64, 96, 128 views. As shown in the images, the traditional Filtered Back Projection (FBP) method introduces significant stripe artifacts, which are not only visually disruptive but also critically compromise diagnostic accuracy in clinical settings, rendering this approach unsuitable for low-dose CT reconstruction tasks.

**Figure 7:**
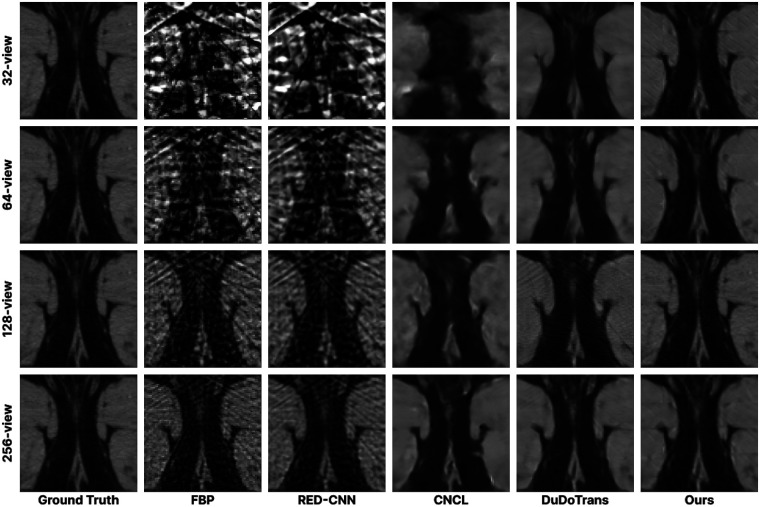
Zoomed ROI of the yellow rectangle in Figure 6. We can see our method not only effectively eliminates artifacts but also significantly enhances detail preservation.

**Figure 8:**
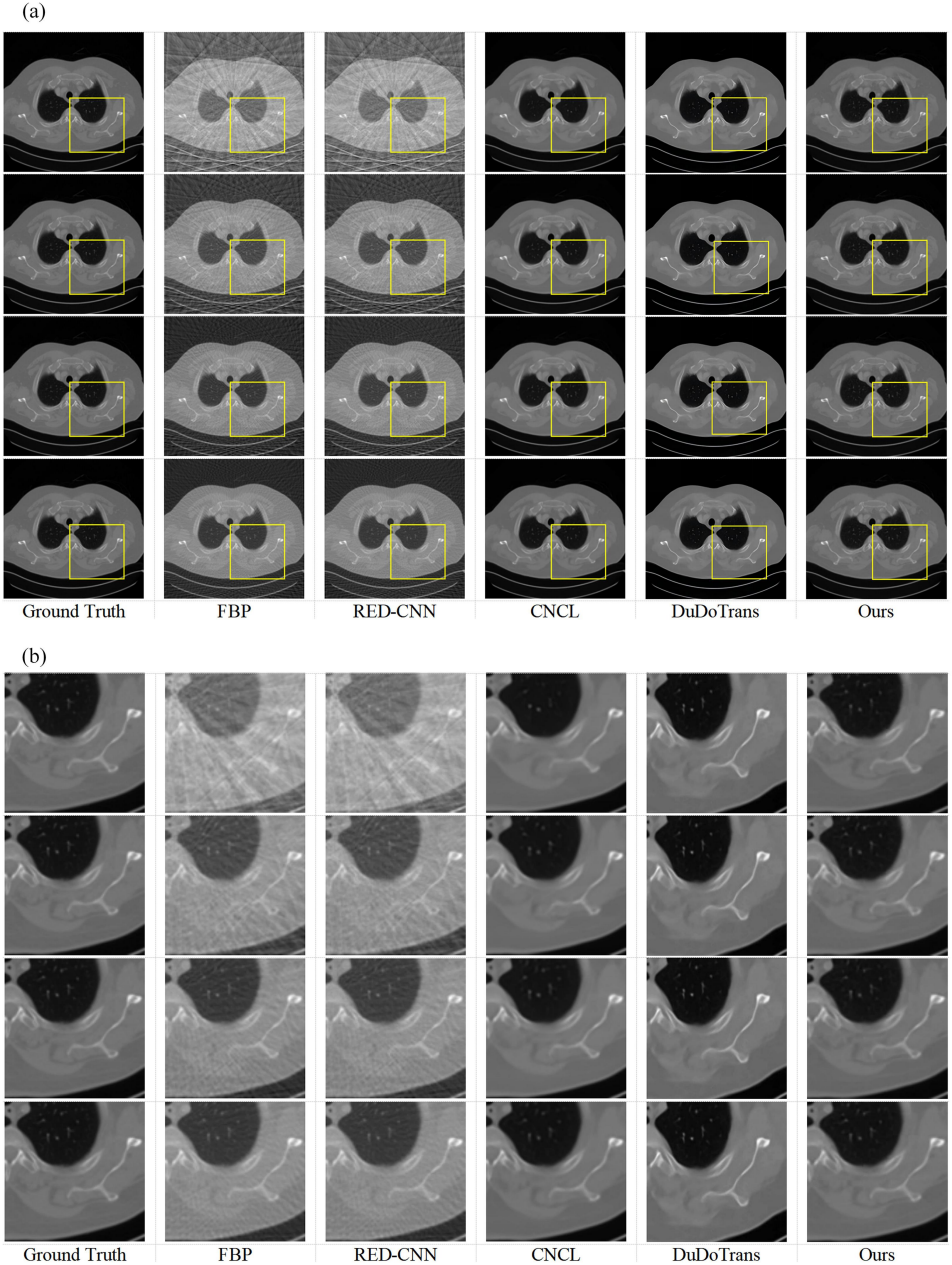
**(a)** visual comparison of different reconstruction methods on representative slices from different anatomical levels. **(b)** The enlarged ROI regions highlight local anatomical details. CDTransNet achieves better artifact suppression and structural preservation than the competing methods across different anatomical levels.

To illustrate the method's performance, one slice per view was selected from the 96-view dataset. Figure 6 shows reconstruction results for four views, comparing different methods. Traditional FBP introduces severe stripe artifacts that degrade visual quality and diagnostic reliability, making it unsuitable for sparse-view CT. In contrast, DL-based methods produce clearer images with clinically acceptable quality. Figure 7 zooms into the yellow box in Figure 6 to highlight detailed differences. To further demonstrate the robustness and generalizability of the proposed method, we expanded the subjective visual comparison by including multiple representative slices from different anatomical levels, as shown in Figure 8. These slices were selected from the test set to cover different anatomical structures and image characteristics. Compared with FBP, RED-CNN, DuDoTrans, and CNCL, the proposed CDTransNet consistently suppresses sparse-view streak artifacts while preserving anatomical structures and edge details across different anatomical levels. In particular, CDTransNet produces clearer structural boundaries and fewer residual artifacts in the enlarged regions, indicating its robust reconstruction performance on different slices.

### 3.3 Quantitative results

**Figure 9:**
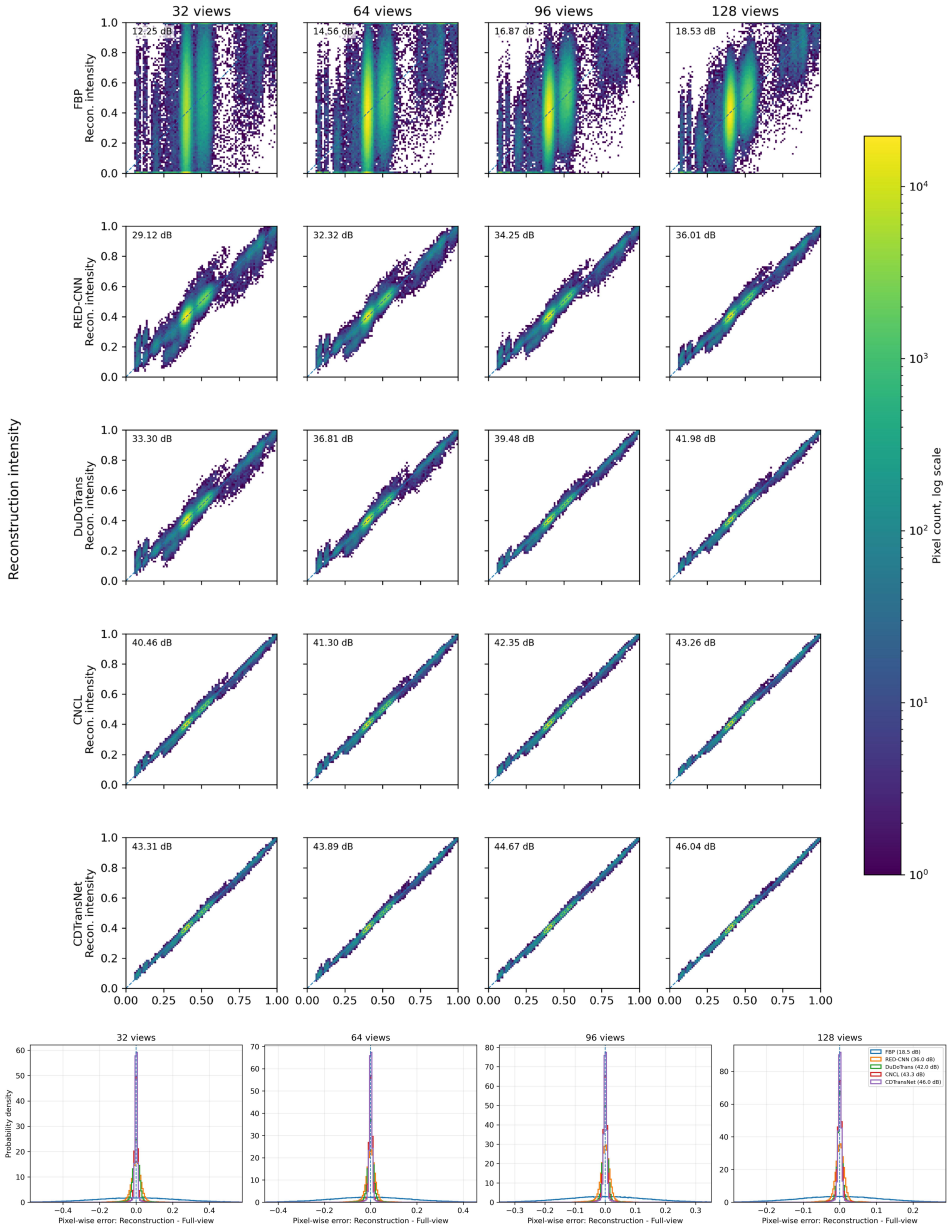
Histogram-based quantitative comparison of different reconstruction methods. The joint histograms illustrate the intensity consistency between the full-view reference images and the reconstructed images, while the error histograms show the pixel-wise residual distributions. CDTransNet produces joint histograms that are more concentrated along the diagonal and error histograms that are more sharply centered around zero, indicating better reconstruction fidelity.

To illustrate the method's performance, one slice per view was selected from the 96-view dataset. To quantitatively compare the relevant methods, we performed a 5-fold cross-validation using the Mayo dataset, employing PSNR, SSIM and NRMSE as the key metrics for image quality assessment. R^2^ was also evaluated between all methods to provide a more comprehensive assessment of reconstruction performance. All the results presented in Table 1, indicate that all methods enhanced the quality of the reconstructed images according to these metrics. Besides, histogram-based quantitative analyses are presented in Figure 9. It shows the joint histograms between the full-view reference images and the reconstructed images under different sparse-view settings. For FBP, the joint histogram exhibits a relatively dispersed distribution, especially under the 32-view and 64-view settings, indicating severe inconsistency between the reconstructed image and the full-view reference. Although RED-CNN and DuDoTrans reduce the dispersion to some extent, their distributions still deviate from the diagonal line due to residual artifacts and intensity bias. For error histograms, the FBP produces a broad error distribution, indicating large reconstruction deviations caused by sparse-view artifacts. RED-CNN and DuDoTrans substantially reduce the error range, but their residual distributions remain wider than those of CNCL and CDTransNet. CDTransNet achieves the narrowest error distribution centered around zero, suggesting that the proposed method introduces less reconstruction bias and achieves more accurate pixel-wise recovery. Notably, our proposed method exhibited superior performance, surpassing the other methods in both metrics. Specifically, as illustrated in Figure 7, our method not only effectively eliminates artifacts but also significantly enhances detail preservation.

**Table 1:** Quantitative comparison of different reconstruction methods under various sparse-view settings.

Methods	32 views				64 views				96 views				128 views			
	PSNR	SSIM	NRMSE	R^2^	PSNR	SSIM	NRMSE	R^2^	PSNR	SSIM	NRMSE	R^2^	PSNR	SSIM	NRMSE	R^2^
FBP	12.180 ± 1.152*	0.348 ± 0.032*	0.246 ± 0.033*	−3.6014	14.770 ± 2.430*	0.456 ± 0.023*	0.183 ± 0.051*	−1.8353	17.201 ± 1.893*	0.538 ± 0.018*	0.138 ± 0.030*	−0.6508	18.874 ± 2.013*	0.598 ± 0.011*	0.114 ± 0.026*	−0.1326
RED-CNN	28.829 ± 1.218*	0.771 ± 0.012*	0.036 ± 0.005*	0.9065	32.332 ± 1.342*	0.890 ± 0.018*	0.024 ± 0.004	0.9549	34.339 ± 1.207	0.918 ± 0.017	0.019 ± 0.003*	0.9707	36.131 ± 1.047*	0.941 ± 0.016*	0.016 ± 0.002*	0.9809
DuDoTrans	33.624 ± 1.426	0.886 ± 0.021	0.021 ± 0.003*	0.9637	37.503 ± 1.846*	0.927 ± 0.030*	0.013 ± 0.003*	0.9826	39.498 ± 1.542*	0.945 ± 0.032*	0.011 ± 0.002*	0.9911	40.802 ± 2.110*	0.955 ± 0.019*	0.009 ± 0.002*	0.9946
CNCL	40.063 ± 1.156*	0.969 ± 0.015*	0.010 ± 0.001*	0.9931	41.781 ± 1.429*	0.978 ± 0.042*	0.008 ± 0.001*	0.9943	42.422 ± 1.262	0.981 ± 0.026	0.008 ± 0.001*	0.9955	43.452 ± 1.029*	0.986 ± 0.012*	0.007 ± 0.001*	0.9964
Ours	43.030 ± 1.015*	0.980 ± 0.027*	0.007 ± 0.001*	0.9965	43.783 ± 1.452*	0.982 ± 0.036*	0.006 ± 0.001*	0.9967	44.846 ± 1.340*	0.985 ± 0.013*	0.006 ± 0.001*	0.9974	45.456 ± 2.011*	0.987 ± 0.018*	0.005 ± 0.001*	0.9981

PSNR, SSIM and NRMSE are evaluated on the test dataset for 32, 64, 96, and 128 projection views. The proposed CDTransNet consistently outperforms existing image-domain and dual-domain baselines across all sampling conditions. Paired t-test between all baseline algorithms and our method was performed with metrics. *p＜0.05.

To investigate the effectiveness of the cross-domain attention mechanism, we replaced the cross-attention module in the model with a conventional multi-head attention operation. This alternative approach directly calculates multi-head self-attention across all input tokens instead of splitting and merging dual-domain data. All other settings remained unchanged, and this experiment is referred to as exp-1. The corresponding results are presented in Table 2. The quantitative evaluation results were presented in Table 3, and a selection of the reconstructed images is displayed in Figure 3. The comparison of GPU memory usage, training time, and model size among different learning-based methods is summarized in Table 4.

**Table 2:** Ablation study of the proposed architecture.

Modules	PSNR	SSIM	NRMSE	Note
BASE	44.846 ± 1.340	0.985 ± 0.013	0.006 ± 0.001	Base model
exp-1	44.695 ± 2.092	0.984 ± 0.075	0.006 ± 0.001	Conventional multi-head attention
exp-2	42.036 ± 2.051	0.981 ± 0.016	0.008 ± 0.002	Without cross attention
exp-3	43.701 ± 2.103	0.981 ± 0.009	0.007 ± 0.002	Replace CFL with MLP
exp-4	43.780 ± 1.624	0.980 ± 0.020	0.006 ± 0.001	Only image loss
exp-5	44.536 ± 1.667	0.984 ± 0.011	0.006 ± 0.002	Both image and sinogram loss
exp-6	44.754 ± 1.806	0.984 ± 0.018	0.006 ± 0.001	Both image and perceptual loss

Each variant removes or replaces a key component of the network, including multi-head attention design, cross-attention module, convolutional fusion layer (CFL), and different loss combinations. Results demonstrate the critical role of cross-attention and dual-domain losses in achieving optimal reconstruction performance.

**Table 3:** Evaluation of cross-domain robustness on datasets with different reconstruction kernels and slice thickness. .

Protocols	32 views			96 views		
	PSNR	SSIM	NRMSE	PSNR	SSIM	NRMSE
1mmB30	43.409 ± 1.132	0.977 ± 0.028	0.007 ± 0.002	44.501 ± 1.421	0.980 ± 0.012	0.006 ± 0.001
1mmD45	42.672 ± 1.211	0.971 ± 0.018	0.008 ± 0.001	43.572 ± 1.047	0.974 ± 0.033	0.007 ± 0.001
3mmB30	43.030 ± 1.277	0.980 ± 0.014	0.007 ± 0.001	44.846 ± 1.291	0.985 ± 0.030	0.006 ± 0.001
3mmD45	42.851 ± 1.209	0.978 ± 0.012	0.007 ± 0.001	44.158 ± 1.147	0.982 ± 0.019	0.006 ± 0.001

The model maintains strong PSNR, SSIM and NRMSE performance across varying clinical imaging protocols (1 mm/3 mm slice thickness and B30/D45 kernels), indicating good robustness and generalizability.

**Table 4:** Comparison of GPU memory usage, training time, and model size with other learning based models.

Method	GPU memory usage	Training time per epoch/s	Model Size (Parameters)
RED-CNN	∼1.5–3 GB	∼20–50	∼1M–1.5M
DuDoTrans	∼10 GB	∼200–300	∼20M–30M
CNCL	∼4–8 GB	∼50–100	∼5M–10M
CDTransNet	∼12 GB	∼356	∼40M

## 4 Discussion

CT technology has become the most widely used auxiliary diagnostic tool in modern clinical medicine. To minimize the potential harm caused by x-ray radiation, the application of sparse-view CT has garnered increasing attention. This study proposed a dual-domain reconstruction method based on a Transformer architecture specifically designed for sparse-view CT imaging in order to reconstruct high-quality CT images from sparse-view sinogram data.

We employed Transformer architecture to overcome the limitations of finite receptive fields inherent in conventional CNN structures. Our method used a parallel dual-domain reconstruction approach, incorporating mixed attention mechanisms to capture latent information between the projection and image domains. Instead of adopting typical Multi-Layer Perceptrons (MLPs) in Transformer models, we introduced a CFL to enable the model to effectively learn long-range dependencies across image patches. Furthermore, we utilized a joint loss function to simultaneously compute reconstruction loss in both domains and perceptual loss in the image domain, thereby preserving more image details. In the following four sections, we evaluated and validated the effectiveness of various components and parameter settings in the proposed model. This included an assessment of the hybrid attention module, evaluation of the convolution fusion layer, verification of the dual-domain loss function's efficiency, and determination of the optimal loss weight parameters. Additionally, we examined the model's robustness across different datasets and analyzed both the computational cost and parameter count required during training and inference. All experiments were conducted on a simulated dataset consisting of 96 views from the 3mmB30 dataset, with PSNR, SSIM and NRMS values calculated for all reconstructed CT images in the test set.

We compared our model with previous SOTA models in which the results are shown in Table 1. From the results, we can find CNCL is better than DuDoTrans. Dual-domain reconstruction frameworks tend to outperform pure image-domain networks because they incorporate physics-based constraints. However, the performance of a specific method is also affected by architecture design, training strategies, data domain, and hyperparameters. Dual-domain does not guarantee universal superiority; effectiveness depends on how well the sinogram branch is adapted to the specific acquisition protocol. DuDoTrans's sinogram encoder generalized poorly to our scans, whereas CNN-based methods are less sensitive to the mismatch.

Our method is better than DuDoTrans although both of them are based on Transformer and domains. Our Cross-Domain TransNet introduces a much stronger, explicitly designed dual-domain interaction mechanism. (1) Our model uses bidirectional cross-attention, not separate domain Transformers. DuDoTrans applies self-attention on each domain separately and relies on implicit mapping. In contrast, we introduce a Cross-Domain Attention Module that includes: synchronized self-attention in each domain and explicit cross-attention. This design forces image-domain features to guide sinogram restoration and vice versa, significantly enhancing both. (2) Progressive fusion in every Transformer block. Each Cross-Domain Transformer Block repeatedly, which splits tokens into two domains, performs domain-specific self-attention, performs cross-attention to exchange feature information and fuses the updated dual-domain tokens. This progressive multi-stage interaction is absent in DuDoTrans. (3) Convolutional Fusion Layer (CFL) replaces the MLP. These designs significantly improved reconstruction quality in Table 1. The histogram-based analysis further confirms that the proposed CDTransNet not only improves global image quality metrics such as PSNR and SSIM, but also reduces pixel-wise reconstruction deviations. The compact joint histogram distribution and the narrow error distribution demonstrate that CDTransNet can better preserve the intensity distribution of full-view CT images while suppressing sparse-view artifacts.

To further explore the impact of dual-domain data interaction on reconstruction quality, we removed the cross-linking in the attention mechanism, calculating self-attention independently for each domain. This experiment is labeled as exp-2, with the results also shown in Table 2. Lastly, to verify the influence of the CFL on the model's performance, the conventional MLP module was replaced with the proposed CFL module in the Transformer architecture. This experiment is referred to as exp-3. We proposed the Cross-Domain TransNet for sparse-view CT image reconstruction, utilizing a joint loss function. The choice of loss functions in DL significantly influenced the image reconstruction process. During our experiments, it became clear that the weights assigned to different loss function terms directly affect the quality of the reconstructed images. Initially, we aimed to establish the relationship between various parameters and the resulting image quality. To identify the optimal weights for each loss term within the joint loss function, experiments were conducted where one parameter was varied while the others were kept constant, thereby determining the best value for each parameter. More importantly, the ablation experiments show that: (1) Exp-1 without domain merging performs similarly to the BASE model because only self-attention is used; the two domains do not interact, so the model cannot leverage additional information. While Exp-2 without cross-attention severely degrades performance, confirming that simple concatenation or self-attention alone cannot effectively integrate projection of image relationships. The significant performance drop in Exp-2 demonstrates that cross-attention is the key operation for meaningful dual-domain interaction, enabling the sinogram features to guide image-space refinement and vice versa. This confirms the reliability and necessity of the cross-attention design in our method.

To further explore the roles of different loss function terms in image reconstruction and assess the effectiveness of the joint loss function, the following experiments were implemented: 1) Using only the image loss, denoted as exp-4.2) Using both image loss and sinogram loss, denoted as exp-5.3) Using both image loss and perceptual loss, denoted as exp-6. The experimental results list in Table 2. When without the dual-domain loss function in the training process, the quality of the image is lower comparing to the results using the dual-domain loss function. To validate the robustness of the proposed method across different datasets, the training and test on images reconstructed using four distinct reconstruction kernels provided by the Mayo dataset were conducted. The experiments were performed with 32 views and 96 views, following the same data preparation process as used for the 3mmB30 dataset. As shown in Table 3 and Figure 3, the proposed method achieved satisfactory evaluation metrics and delivered visually appealing results across all four datasets. Besides, as we can see the CDTN results appear to exhibit patch-edge artifacts in Figure 7. The mild patch-edge artifacts are caused by the fixed-size patch embedding used in the Transformer encoder. When anatomical structures span across patch boundaries, the global self-attention may capture long-range relationships, but local continuity between adjacent patches is not fully preserved, especially at higher sparsity levels. The proposed CFL module is designed to alleviate this issue by restoring spatial continuity and performing local feature fusion after attention. While the CFL significantly reduces patch-edge inconsistencies, some residual artifacts can remain under extremely sparse-view settings.

Nonetheless, this study has certain limitations. Firstly, the model heavily relies on high-quality paired training data with accurate ground-truth reconstructions. In practice, obtaining such paired data is challenging, especially for sparse-view CT where high-dose full-view reconstructions may not be available or feasible due to radiation concerns. Secondly, our current experiments use simulated projections because raw clinical projection data are not publicly available for the benchmark dataset used in this study. We acknowledge that simulation may not fully capture detector noise characteristics, system blur, scatter, or calibration imperfections. Therefore, a domain gap between simulated and real measurements may exist. We will include a discussion on this limitation. Thirdly, the dual-domain design, combined with attention-based mechanisms and the proposed Convolutional Fusion Layer (CFL), leads to increased memory consumption and inference time. This may hinder the deployment of the model in time-sensitive or resource-constrained environments, such as portable imaging devices or point-of-care systems. Future work could explore lightweight model variants or optimization techniques, such as knowledge distillation or model pruning, to reduce computational costs while maintaining performance.

## 5 Conclusion

In conclusion, we conducted extensive qualitative and quantitative analyses on publicly available datasets, comparing the proposed method with traditional approaches, single-domain DL methods, and state-of-the-art dual-domain DL techniques. Our approach not only achieves high quantitative metrics but also delivers superior visual results. It effectively preserves the structure and texture of CT images while removing noise and artifacts, even when the projection data is under-sampled. Finally, through multiple ablation experiments, we validated the effectiveness of the model components and identified the optimal parameter settings.

## Data Availability

The original contributions presented in the study are included in the article/Supplementary Material, further inquiries can be directed to the corresponding authors.
