# Supplementary material for: Cross-Domain TransNet for sparse-view CT reconstruction
Source: Front Nucl Med. 2026 Jul 6;6:1711533. doi: 10.3389/fnume.2026.1711533 (PMC13386490; doi:10.3389/fnume.2026.1711533)
Supplement: Supplementary file 1 [file Table1.docx]

Supplementary Material

# S1: Datasets

The training and testing of the method used publicly available data from the 2016 NIH-AAPM-Mayo Low Dose CT Grand Challenge dataset, denoted as mayo dataset. This dataset includes four sets of images from 10 patients, which were reconstructed using the B30 reconstruction kernel and D45 reconstruction kernel for 1mm and 3mm slice thicknesses. These are denoted as 1mmB30, 1mmD45, 3mmB30, and 3mmD45 datasets. The mayo dataset was acquired using Siemens Somatom Definition AS+ and Definition Flash scanners operated in single-source mode. Besides, the imaging geometry is characterized by a source-to-isocenter distance (SOD) of 570 mm and a source-to-detector distance (SDD) of 1040 mm. All projection data are provided in the DICOM-CT-PD format, which extends the conventional DICOM standard to include raw projection data and detailed scan geometry.

As the same patient and the same part of the body of different sparse-view projection data is difficult to obtain, this study uses the simulation method to generate different doses of projection data. The noise was simulated by adding Poisson noise to the projection data of conventional doses.

Specifically, for this study, a total of 2378 3mm slices reconstructed using the B30 reconstruction kernel were chosen from 10 patients, with a resolution of 256×256. Out of these, 237 slices were randomly selected for testing, while the remaining 2141 slices were used for training and validation for the models.

A scanning geometry with a fan-beam X-ray source comprising 800 detector elements was simulated. In our experiments, four sparse-view scenarios were simulated, corresponding to different numbers of views, which were set to view = [32, 64, 96, 128]. Sparse-view CT images were initially reconstructed from the sparse-view sinograms using the Filtered Back Projection (FBP) algorithm implemented with the ASTRA Toolbox. A Ram-Lak filter was adopted during the reconstruction process under fan-beam geometry. The reconstructed CT images had a matrix size of 256 × 256 pixels. The FBP reconstruction was only used to generate the initial reconstruction input for the proposed network. These views were uniformly sampled within a 360-degree circle around the patient. To simulate photon noise in the numerical experiments, mixed noise was added to the sinogram data, consisting of 5% Poisson noise with an intensity of ${5e}^{6}$.

# S2: Methods

**Cross-Domain TransNet Architecture**

We propose a sparse-view CT reconstruction method based on the Vision Transformer architecture that incorporates a dual-domain cross-attention mechanism. The main idea is to progressively extract potential relationships between image domain and projection domain data, thereby complementing and enhancing the recovery of both domains. By integrating prior knowledge from the image domain, noise and artifacts in the reconstructed images can be effectively suppressed, leading to improved image quality. Furthermore, the computational burden of the reconstruction process can be alleviated through exploiting the inherent sparsity or redundancy in the projection domain data. The development of appropriate algorithms that synergistically fuse these complementary sources of information can significantly improve the robustness of the reconstruction against noise and data incompleteness.

The attention module within the Transformer architecture is highly flexible, capable of dynamic and global modeling, with minimal assumptions about data structure. The above advantages make it broadly applicable to general relationship modeling. Leveraging the flexibility, a cross-attention module that facilitates interaction between projection domain and image domain data was introduced. In our approach, a hybrid interaction scheme is designed to perform both self-attention and cross-attention operations on features for the projection and image domains. Specifically, self-attention operations focus on extracting features within each respective domain. While cross-attention operations enable communication between the domains, fusing information from the projection and image data effectively.

The overall framework of the method proposed is primarily composed of three parts: initial Patch Embedding operation, stacked Cross-Domain Transformer modules, and Patch Merging operation. Specifically, the proposed CDTransNet consists of six stacked Cross-Domain Transformer blocks. Each Transformer block employs eight attention heads with an embedding dimension of 256, while the hidden dimension of the feed-forward network is set to 1024. Firstly, a sparse-view CT image is initially reconstructed by FBP from a sparse-view sinogram. Then, the sparse-view sinogram and the sparse-view CT image are put into the model. Subsequently, Patch Embedding module is applied to the dual-domain data, followed by the addition of positional encoding. Subsequently, the Patch Embedding module is applied to the dual-domain data. Specifically, both the image-domain CT reconstruction and projection-domain sinogram are divided into non-overlapping patches of size 8 × 8 and projected into embedding tokens. Positional encodings are then added to preserve spatial information. The resulting embeddings are concatenated into a token input for the cross-domain Transformer module. After the data fusion and recovery within the Cross-Domain Transformer blocks, the output tokens are split. Then, the Patch Merging Module is performed to obtain the final reconstructed image.

**Cross-Domain Attention Module**

The Cross-Domain Transformer Block takes the concatenated dual-domain tokens as input. It applies layer normalization to standardize the tokens and prevent data offset during training. The cross-attention module then splits the dual-domain tokens into single-domain tokens, where self-attention within each domain is synchronized with cross-attention between the domains. The multi-head self-attention calculation operation establishes global correlations within each data domain. This operation allows our model to understand domain-specific feature information better and capture long-distance dependencies, which are crucial for extracting key features. The cross-attention computation facilitates effective interaction and fusion of feature information between the different domains. Besides, it enhances the representation of projection and image features in CT reconstruction and enabling information sharing between the two domains.

After the attention operation, CT image and sinogram features are concatenated again as a dual-domain token, which is then added to the residual connection of the input. Next, the output of the cross-attention module undergoes a normalization operation to standardize the feature representations of the two data domains. In which, it helps to ensure that the feature distributions across domains are similar. Finally, the output is processed through a convolutional fusion layer (CFL).

The self-attention operation may not capture and fuse information of the different data domains, which limits the performance of the model. Cross-attention operation could allow information fusion between CT image data and projection data, which helps to improve the accuracy and reliability of image reconstruction. As shown in Figure 3, linear projections are applied to each of them to generate the queries (*Q*), keys (*K*), and values (*V*) required for the attention operation in the cross-attention operation. Using $Q_{\text{ct}}$, $K_{\text{ct}}$, $V_{\text{ct}}$ to represent the query, key, and value for the CT image domain, and $Q_{\text{sino}}$, $K_{\text{sino}}$, $V_{\text{sino}}$ for the projection domain. We concatenate $K_{\text{ct}}$ and $K_{\text{sino}}$ to calculate $K_{\text{cross}}$, and concatenate $V_{\text{ct}}$and $V_{\text{sino}}$ to calculate $V_{\text{cross}}$, the cross-attention operation can be defined as follows:

$$\begin{aligned} \text{Attention}_{\text{ct}}=\text{Softmax}\left( \frac{Q_{\text{ct}}K_{\text{cross}}^{T}}{\sqrt{d}} \right)V_{\text{cross}} ,\#\left( 1 \right) \end{aligned}$$

$$\begin{aligned} \text{Attention}_{\text{sino}}=\text{Softmax}\left( \frac{Q_{\text{sino}}K_{\text{cross}}^{T}}{\sqrt{d}} \right)V_{\text{cross}} ,\#\left( 2 \right) \end{aligned}$$

$$\begin{aligned} K_{\text{cross}}=\text{Concat}\left( K_{\text{ct}}, K_{\text{sino}} \right),\#\left( 3 \right) \end{aligned}$$

$$\begin{aligned} V_{\text{cross}}=\text{Concat}\left( V_{\text{ct}}, V_{\text{sino}} \right),\#\left( 4 \right) \end{aligned}$$

where *d* represents the dimension of keys, $\text{Attention}_{\text{ct}}$ and $\text{Attention}_{\text{sino}}$ respectively denote the attention scores of image domain and projection domain.

**Convolutional Fusion Layer**

Inspired by Vision Transformer (ViT), a common mechanism in Transformers for image processing involves dividing the input image into fixed-sized patches. Then, it transforms these patches into 1D vectors to represent images as continuous word piece vectors linearly. However, fixed-sized patch representations can lead to information loss. Since the patches are arbitrarily divided, objects, features, or structures that may span multiple patches cannot be considered. This limitation arises because the self-attention mechanism and fully connected layers treat each patch as an independent entity. One approach to mitigate this issue is to use a cascaded Transformer with patches of varying sizes through a sliding window. However, this will result in a significant increase in computational costs typically. Additionally, while self-attention does establish global relationships, these relationships are pairwise and depend solely on positional encodings to capture the global structure.

In contrast, CNNs utilize convolutional layers and max pooling to achieve a degree of translation invariance. To fully leverage the capabilities of CNNs in local feature extraction, translation invariance, and structural preservation, we proposed a CFL Module to replace the last MLP layer in the Transformer, enhancing structural preservation and recovery across patches. The structure of the CFL is shown in Figure 4. The proposed CFL consists of two consecutive convolutional layers with kernel size 3 × 3, followed by GELU activation functions. The convolutional operations are introduced to enhance local structural preservation and improve continuity across neighboring patches. In this module, the “Token-to-Image” layer refers to the operation that reshapes the 1D patch tokens back into a 2D spatial feature map before applying convolution. Since Transformer patch embeddings flatten image regions into vectors, spatial structure is lost. In the CFL, we first reproject tokens to their original spatial arrangement, restoring the 2D grid, which then allows the convolutional layers to effectively capture local continuity and anatomical structure.

**Dual-Domain Loss Function**

This work applied a dual-domain loss function to achieve better reconstruction results. The dual-domain loss function comprises three components: image reconstruction loss, image perceptual loss, and reconstruction projection consistency loss.

**Image Reconstruction Loss**

Mean Squared Error (MSE) loss is widely used for measuring the difference between model output images and reference images as a standard method. By minimizing the MSE loss, image reconstruction loss helped ensure that the reconstructed images produced by the model closely match the reference images at pixel level. The image reconstruction loss denoted as $L_{\mathrm{IMG}}$, is computed as follows:

$$\begin{aligned} L_{\text{IMG}}=\text{MSE}\left( \hat{x}_{\text{IMG}},x_{\text{IMG}} \right),\#\left( 5 \right) \end{aligned}$$

where $\hat{x}_{\text{IMG}}$ represents the reconstructed image and $x_{\mathrm{IMG}}$ represents the corresponding reference image.

**Image Perception Loss**

By introducing perceptual loss, a deeper level of feature constraint is applied to image reconstruction. Perceptual loss calculates the distance in the deep feature space between the reconstructed image and the reference image, denoted as $L_{\text{VGG}}$. In this study, a pre-trained VGG-19 model was employed as a feature extractor. The VGG-19 network consists of 16 convolutional layers and 3 pooling layers, the output of the 16th layer is selected as the deep feature extraction result, denoted as VGG (·). The calculation of image perceptual loss is as follows:

$$\begin{aligned} L_{\text{VGG}}=MSE\left( \text{VGG}\left( \hat{x}_{\text{IMG}} \right),VGG\left( x_{\text{IMG}} \right) \right),\#\left( 6 \right) \end{aligned}$$

where $\hat{x}_{\text{IMG}}$ represents the reconstructed image and $x_{\mathrm{IM}G}$ represents the corresponding reference image.

**Reconstruction Projection Consistency Loss**

In addition to the commonly used image reconstruction loss and image perceptual loss mentioned above, we designed a reconstruction projection consistency loss, considering the characteristics of dual-domain mixed training in our proposed method. This loss is calculated by measuring the MSE between the projection data generated during the model training phase and the projection data from standard dose projections, both of which are produced simultaneously with the final reconstructed image. This loss is denoted as $L_{\text{SINO}}$, and the calculation is as follows:

$$\begin{aligned} L_{\text{SINO}}=MSE\left( \hat{y}_{\text{SINO}},y_{\text{SINO}} \right),\#\left( 7 \right) \end{aligned}$$

where $\hat{y}_{\text{SINO}}$ represents the restored sinogram and $y_{\mathrm{SINO}}$ represents the normal dose sinogram.

**Dual-Domain Loss**

In summary, the combined loss function employed in this study is composed of the three loss functions, and it utilizes three hyperparameters, denoted as $\lambda_{\text{IMG}}$,$\lambda_{\text{VGG}}$, and $\lambda_{\text{SINO}}$, to control the weighting of each loss component. This results in the final objective function, denoted as $L_{\text{total}}$ , calculated as follows:

$$\begin{aligned} L_{\text{total}}=\lambda_{\text{IMG}}L_{\text{IMG}}+\lambda_{\text{VGG}}L_{\text{VGG}}+\lambda_{\text{SINO}}L_{\text{SINO}},\#\left( 8 \right) \end{aligned}$$

The training method using a joint loss function allows the proposed model to function as an end-to-end DL framework, which continuously learns the characteristics of projection data, CT images, and the implicit relationships between the dual domains during training by minimizing the loss function. The design of this dual-domain loss function reflects a comprehensive approach to balancing task performance and model robustness, combining different loss terms to meet the requirements of accuracy and robustness effectively.

Through the iterative application of backpropagation and optimization techniques, the model’s weight parameters were fine-tuned to minimize the overall joint loss function. This process enables the model to optimize itself on the training data gradually, enhancing its adaptability and performance for complex tasks over time. In our experiments, we validated the effectiveness of the joint loss function, evaluate the impact of different loss functions and their weights on the final reconstructed image quality. For specific experimental results, please refer to Section V, Part D.

## S3: Experimental Setup

In this section, we implemented the proposed method in Python using the PyTorch library and employed the Adam optimizer to optimize all model parameters with a setting of $\left( \beta_{1},\beta_{2} \right)=(0.9, 0.999)$. The Adam optimizer was adopted with an initial learning rate of 1×10⁻⁴. The weights for the three loss functions in the objective function were configured as $\left\{ \lambda_{\mathrm{IMG}}, \lambda_{\mathrm{VGG}}, \lambda_{\mathrm{SINO}} \right\}=\{1, 0.001, 0.01\}$. More details were also shown in Supplementary Materials S2.

To evaluate the reconstructed images quantitatively, we adopted the Peak Signal-to-Noise Ratio (PSNR) and the Structural Similarity Index (SSIM) as assessment metrics. We conducted a total of 300 training epochs, with a batch size of 1, and a learning rate of 0.0001. The batch size was set to 1 due to the high GPU memory consumption introduced by the dual-domain Transformer architecture and high-resolution CT inputs. All the networks were implemented on an Ubuntu 22 system with an Intel(R) Core i7-6700K CPU, 64 GB RAM, and a GTX TITAN X GPU with 12GB memory.
